# The Role of CD56 in Thyroid Fine Needle Aspiration Cytology: A Pilot Study Performed on Liquid Based Cytology

**DOI:** 10.1371/journal.pone.0132939

**Published:** 2015-07-17

**Authors:** Tommaso Bizzarro, Maurizio Martini, Carla Marrocco, Donato D’Amato, Emanuela Traini, Celestino Pio Lombardi, Alfredo Pontecorvi, Guido Fadda, Luigi Maria Larocca, Esther Diana Rossi

**Affiliations:** 1 Division of Anatomic Pathology and Histology, Università Cattolica del Sacro Cuore, “Agostino Gemelli” School of Medicine, Rome, Italy; 2 Division of Endocrine Surgery, Università Cattolica del Sacro Cuore, “Agostino Gemelli” School of Medicine, Rome, Italy; 3 Division of Endocrinology, Università Cattolica del Sacro Cuore, “Agostino Gemelli” School of Medicine, Rome, Italy; University of Utah Health Sciences Center, UNITED STATES

## Abstract

**Background:**

Fine needle aspiration Cytology (FNAC) fulfills a reliable role in the evaluation of thyroid lesions. Although the majority of nodules are quite easily diagnosed as benign or malignant, 30% of them represent an indeterminate category whereby the application of ancillary techniques (i.e. immunocytochemistry-ICC and molecular testing) has been encouraged. The search for a specific immunomarker of malignancy sheds light on a huge number of ICC stains although none of them attempt to yield 100% conclusive results. Our aim was to define in a pilot study on thyroid FNAC whether CD56 might be a valid marker also in comparison with HBME-1 and Galectin-3.

**Materials and Methods:**

Inasmuch as this is the largest pilot study using only liquid based cytology (LBC), we selected all the cases only in the categories of benign nodules (BN) and positive for malignancy (PM) for validation purposes. Eighty-five consecutive (including 50 PM and 35 BN) out of 950 thyroid FNACs had surgical follow-up. The ICC panel (HBME-1, Galectin-3 and CD56) was carried out on LBC and histology.

**Results:**

All BNs and PMs were histological confirmed. CD56 was negative in 96% of the PM while 68.5% of the BNs showed cytoplasmic positivity for this marker, with an overall high sensitivity (96%) but lower specificity (69%). In specific, our 96% of the PMs did not show any follicular cell with CD56 expression. Different ICC combinations were evaluated showing that the panel made up of CD56 plus HBME-1 and Galectin-3 had the highest sensitivity (98%) and specificity (86%).

**Conclusions:**

Our pilot study suggests that CD56 may be a good marker for ruling out PTC and its variants. The low specificity suggests that an immunopanel including also HBME-1 and Galectin-3 could obtain the highest diagnostic accuracy in thyroid lesions. Our results suggest that CD56 may be a feasible additional marker for identifying malignancies also in the FNs and SMs.

## Introduction

Thyroid nodules represent a frequent finding in the general population, including both non-neoplastic and neoplastic lesions with a total rate of malignancy estimates at around 5% [1–3].

The assessment of different entities underscores the need to discriminate those nodules that require surgery from those that can be safely followed-up. In this perspective, fine needle aspiration cytology (FNAC) is the most accurate and cost-effective screening method relying on a 95% diagnostic accuracy [1–5]. Above all malignancies, papillary thyroid carcinoma (PTC) is the most frequent histotype with a prevalence ranging from 70 to 85% of all the thyroid cancers [1–3].

Albeit the PTC diagnosis is straightforward in the majority of cases and it hinges on specific morphologic features (i.e. papillary structures, nuclear grooves, nuclear inclusions and ground glass nuclei), however there are samples with low amount of follicular cells or with the evidence of subtle and suspicious malignant features which may hinder the attempts for a definitive diagnosis of malignancies (6–12). As a result, a conclusive PTC diagnosis cannot be render in approximately 25%-30% of the thyroid FNAC justifying either the diagnosis of Follicular Neoplasms (FN) or suspicious for malignancies (SM) [6, 10–11].

As far as it concerns, these latter categories have been a source of contentious debate as evidenced by the number of publications discussing their diagnostic efficacy and implications in the recommended and suggested management of patients [3, 4, 6–9].

Inasmuch as either the distinction between PTCs versus hyperfunctional lesions or the recognition of malignancies in the FN (in a range of 25%-30% FNs) may lead to some relevant pitfalls, a huge number of papers pointed out the invaluable role of ancillary techniques including both immunocytochemistry (ICC) and molecular panels in refining and empowering the diagnostic accuracy of morphological specimens especially in the latter category [12–18].

To ascertain whether a "key marker of malignancy" can be found, a growing number of immunomarkers, tested as single or as panels, have been carried out with a considerable variability of results and among them, HBME-1, Galectine-3 and Cytokeratin-19 have been reported to have the highest diagnostic accuracy [12–20].

Consistent with our previous feasible and reliable experiences with ICC on liquid based cytology (LBC), here we reported a pilot study including a series of all the 85 thyroid cyto-histological prospective cases with the evaluation of CD56, an isoform of N-CAM [12, 15–16]. This neural cell adhesion molecule, which was demonstrated to be expressed in normal and benign thyroid tissue and remarkably decreased in papillary thyroid carcinoma (PTC), was quite exclusively reported and studied on histological thyroid series speculating also that CD56 might be further involved in the activation of epithelial mesenchymal transition and modulation of genes regulating metastases [21–26]. We analyzed its possible role as a new valid marker in all the cases belonging to the two FNAC groups of benign and malignant thyroid lesions processed only with LBC. In addition we discuss the role of the immunopanel made up of HBME-1, Galectin-3 compared with CD56 in the same cytological categories.

## Materials and Methods

**Fig 1:**
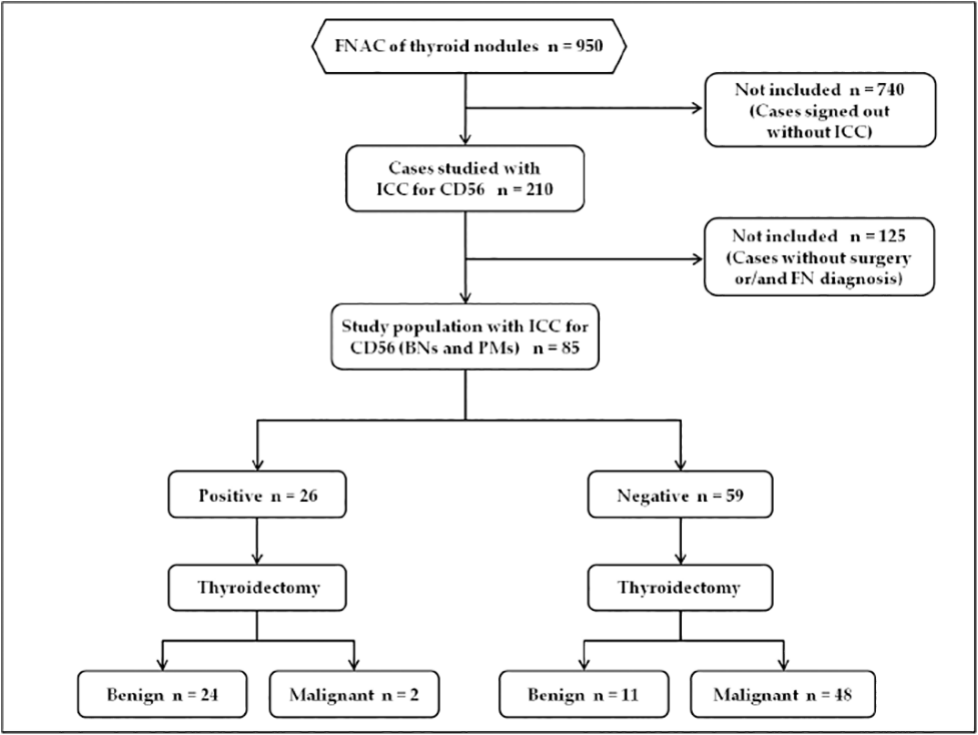
STARD flow diagram of study population.

**Fig 2:**
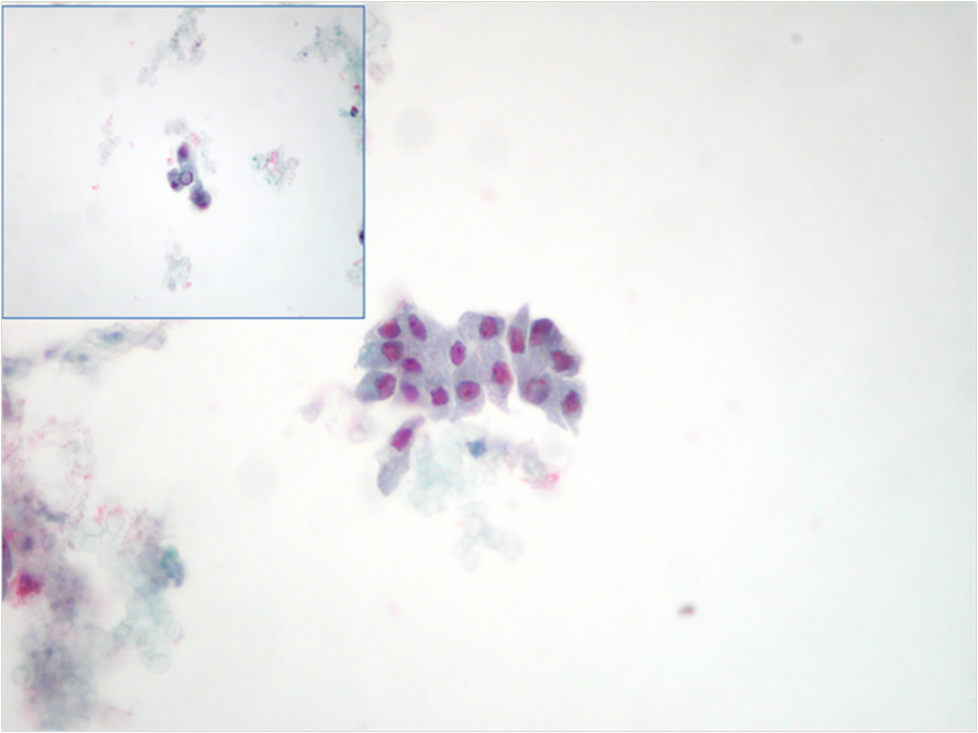
Details of a thyroid lesion diagnosed as positive for malignancy favoring papillary thyroid carcinoma on LBC, in the upper left a detail of nuclear inclusion (PAPx400).

Our patient population included the prospective analysis of all the 950 cytological cases retrieved from the files of the Division of Anatomic Pathology and Histology of the Catholic University, “Agostino Gemelli” Hospital in Rome between May and November 2014. Based on the diagnostic intents of our evaluation, each of the authors had access to patient identifying information at any time. Furthermore for diagnostic purposes 210 out of the global cohort of 950 thyroid FNAC showed morphological details which were additionally studied with the application of the immunopanel made up of HBME-1, Galectin-3 and CD56 (Fig 1). Inasmuch as this pilot study wants to evaluate the diagnostic value of CD56, we referred and included only all the cases belonging to the two thyroid categories diagnosed as benign nodules-BN(35 cases) and as positive for Malignant Neoplasms-PM (50 cases, Fig 2) with histological follow-up in the reference period.

The nodules were identified with sonographic guidance (US) mostly performed by surgeons and endocrinologists (ET, CPL, AP). The nodules size ranged from 4 mm to 70 mm were analyzed and discovered during routinely US thyroid check-up performed in the “Centre for Thyroid Diseases” of the Departments of Endocrinology and Endocrine Surgery of our hospital. Two passes for each lesion were usually performed with 25 to 27 G needles without rapid adequacy assessment of the material and all the cases were entirely processed only with LBC method-Thin Prep 5000 (Hologic Co., Marlborough, MA).

Regarding the use of LBC method, all the patients have been appropriately informed about the aspiration method and a written informed consent was signed by all of them. Our study followed the tenets of the Declaration of Helsinki and we received the internal department (Department of Anatomic Pathology and Histology) ethics approval for the study. We need to specify that we followed the general and normal ethic statement of our University (Catholic University) as long as this is an ICC study used for diagnostic purposes which do not need any ethical university reviewer board approving the study in our institution but only the one from our department. As previously reported in some of our papers, the aspirated material was completely fixed with the hemolytic and preservative solution Cytolite after rinsing the needle in this solution. The resulting LBC slide is fixed in 95% methanol and stained with Papanicolaou while the remaining material is stored in the Preservcyt solution to be used for the preparation of additional slides for further investigations (including both ICC and molecular analysis). All the details about LBC method and ancillary techniques have been described in our previous papers [12, 15, 16].

The adequacy was reported accordingly to the British RCPath classification; in fact the lower limit for the adequacy for each sample was established in six groups of thyroid follicular epithelial cells within the submitted LBC slide and each of these groups with at least 10 well-visualized epithelial cells [27].

Specifically, the cytological cases in the reference period were classified according to the New Italian Working Group SIAPEC-IAP classification [11]. As reported in the new classification system the categories are defined as follows: TIR 1 including: inadequate and TIR1C cystic-hemorrhagic lesions; TIR2: benign nodules-BN; TIR3A: Follicular Neoplasm (low-risk indeterminate lesions); TIR3B Follicular Neoplasm (high-risk indeterminate lesions); TIR4: Suspicious of malignancy-SM; TIR5: positive for malignant neoplasm-PM. For the reader’s convenience apart from the definitions of TIR3A (increased cellularity with numerous microfollicular structures in a background of poor colloid amount) and TIR3B (characterized by a high cellularity in a monotonous and repetitive microfollicular / trabecular arrangement, with scant or absent colloid), the remaining categories did not result in any change from the previous classification system [28].

Although the cytological cases were classified according to the New Italian Working Group SIAPEC-IAP classification, the majority of categories overlapped with the diagnoses adopted by the Bethesda System for Reporting Thyroid Cytopathology [6]. In fact the cytological diagnoses of benign lesions, suspicious for malignancy and positive for malignancy share identical features in both systems; indeed the TIR3A corresponds to AUS/FLUS (Atypia of undetermined significance-AUS/Follicular lesion of undetermined significance-FLUS) whilst TIR3B to FN-Follicular Neoplasm [6].

For the reference period, our entire cytological series included the following distribution of thyroid diagnoses: 6.5% TIR 1 (non diagnostic plus cystic cases); 79% TIR 2; 4.3% TIR 3A; 6% TIR3B; 1.7% SM and 2.5% PM. All the cytological and histological sections were evaluated by two expert pathologists (EDR and GF) and those cases whose interpretation was equivocal were submitted to the diagnostic judgment of the other pathologists until a final agreement was achieved.

### Immunocytochemical Analysis

As described in detail in our previous papers, ICC staining were carried out with the avidin-biotin peroxidase complex on LBC slides obtained from the stored material and with the following antibodies: HBME-1 (Dako, Denmark, 1:100 dilution), Galectin-3 (Ventana, USA, 1:100 dilution) and CD56 (Ventana, USA, 1:100 dilution) [12,15,16]. In detail, each antibody was carried out on different slides obtained from the stored material with a total of 3 additional ICC slides (one per each immunomarker). The ICC method was performed as previously described [12,15, 16]. The positivity was assessed, for each cytological case, when at least 30% of the total epithelial-follicular (for benign lesions) and/or malignant cells (for PM cases) showed moderate or strong cytoplasm positivity. According to our previous experiences in the field of ICC application on LBC, this arbitrary ICC cut-off (30% of the nodular cells) was established based on the histological diagnoses and on the purpose of reducing the number of false positive and false negative results which might be obtained when a 10% expression cut-off is adopted or in cases with faint and/or weakly focal positivity [12, 15–16]. We did not differentiate between moderate and strong positivity as long as they were both considered positive without any further semiquantitative analysis.

Galectin-3 and CD56 displayed cytoplasm staining; HBME1 staining showed both cytoplasm and membranous positivity. Positive controls were represented by mesothelioma cells for HBME-1, histiocytes and macrophages for Galectin-3 and CD56 positivity. All our negative controls were defined by lymphocytes identified in most of the thyroid slides. For comparison, a paraffin block from each of the corresponding histological resection specimens of PTCs and BNs were used for the immunohistochemical (IHC) evaluation. The IHC analysis did not show any cyto-histological discordant yield among the analyzed cases; in fact both preparations (cytological and correspondent histological samples) were concordantly positive or negative using our cut-off. Moreover, the pathologists were blinded to the cytological and subsequent histological diagnosis of each case when they evaluated ICC slides on LBC.

### Histology

Fifty cases underwent surgery based on the cytological diagnoses of malignancy and the 35 benign cytological cases based on the compressive and functional symptoms of nodular goiter. The surgical specimens were fixed in 10% buffered formaldehyde, embedded in paraffin and the 5 micron-thick sections were stained with haematoxylin-eosin. An extensive search of lymph nodes was performed as long as all the fibro-adipose tissue close to the thyroid gland was included for their research. The diagnosis of PTC was based on the presence of true papillary structures and the distinctive nuclear features whereas the diagnosis of FVPC relied upon the detection of the nuclear features of PTC in multiple foci within the tumor including both encapsulated and infiltrative variants. The diagnosis of Tall cell variant was characterized by predominance of neoplastic cells whose heights were at least three times their widths and with classical PTC nuclear features. All the cases were classified according to the seventh edition of the tumor-node-metastasis-based staging system recommended by the American Joint Commission on Cancer (AJCC) [29].

### Statistical Analysis

Statistical analysis was performed by using a commercially available statistical software package (SPSS23, Chicago, IL, USA) for Windows (Microsoft, Redmond, Washington, USA). Comparison of categorical variables was performed by chi-square statistic, using the Fisher’s exact test when appropriate. A p value less than 0.05 has been considered significant. The descriptive statistic, detailed in the result section, was worked out for each immunomarker alone, for the immunopanel made up of all the three HBME-1, Galectin-3 and CD56 as well as for any different combination of two of them included also in the supplementary material (S1 Table)

## Results

As highlighted in the material and Methods section and Table 1, a total of 210 out of all the 950 thyroid FNACs performed in our hospital were analyzed with the additional application of an ICC panel made up of HBME-1, Galectin-3 and CD56 during the study period (from May to November 2014). According to the intents of our research project of CD56 validation in a pilot study on LBC thyroid cases, in the present paper we discussed and considered only the two thyroid categories diagnosed as benign nodules-BN and positive for Malignant Neoplasms-PM (Fig 2) in the same reference period.

**Table 1:** Clinicopathologic features of 85 thyroid lesions with BN and PM diagnoses and histological follow-up.

VARIABLE	BN	PM
**N° Patients**	35	50
**M/F**	11/24	16/34
**Age**	17–84	20–89
**Nodule Size (mm)**	6–60	4–70
**Histological Diagnosis**		
**Goiter**	35	0
**PTC**	0	25
**FVPC°**	0	21
**TCV**	0	4
**Lfn Mets**	0	15
**Extrathyroid Infiltration**	0	8
**Multifocality**	0	20

BN: Benign Nodule; PM: Positive of Malignancy favoring Papillary Thyroid cancer; M/: male/female; Lfn Mets: Lymph nodes metastases; PTC: papillary thyroid carcinoma; FVPC: follicular variant of PTC; TCV: Tall cell variant of PTC; °FVPCs included 11encapsulated and 10 infiltrative variants.

We found 85 (35 BNs and 50 PMs) cytological cases with surgical follow-up (Fig 1) including 30 male and 55 female patients with a median age of 45y/o (Table 1).

All cytological diagnoses were confirmed at histology. In details, BN outcome resulted in 35 nodular goiters while the 50 PMs resulted in 25 PTC, 21 Follicular variant of PTC (FVPC) and 4 Tall cell variant (TCV). All the clinical and pathologic features of the study patients are summarized in Fig 1. For the FVPCs, we diagnosed 11 encapsulated and 10 infiltrative FVPCs.

Table 2 shows the expression of each single immunomarker for the two categories.

**Table 2:** CD56, HBME-1 and Galectin-3 staining score in the 85 BN and PM diagnoses with histological follow-up. Positive and negative expression for each antibody among the cases.

	**CD56**		**HBME-1**		GALECTIN-3	
	Positive	Negative	Positive	Negative	Positive	Negative
**Benign (35)**	24 (68,5%)	11 (31,5%)	8 (22,8%)	27 (77,2%)	10 (28,5%)	25 (71,5%)
**Malignant (50)**	2 (4%)	48 (96%)	45 (90%)	5 (10%)	48 (96%)	2 (4%)

**Fig 3:**
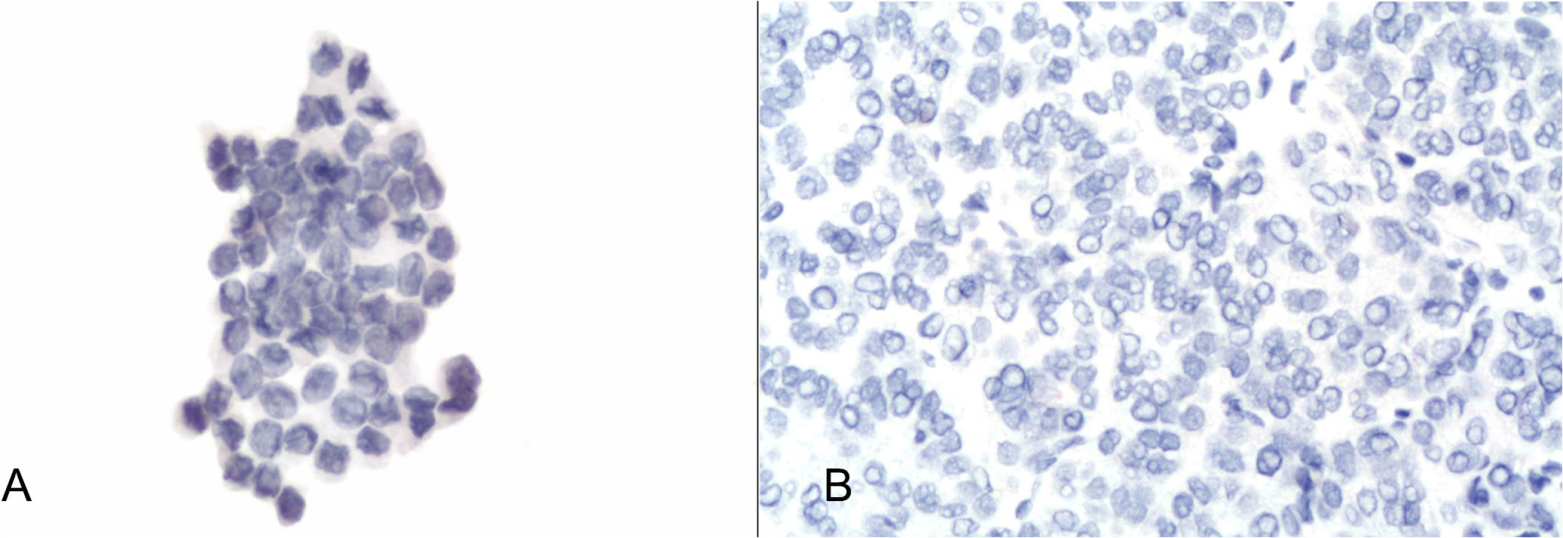
a) Details of CD56 negativity in the same case of Fig 1 (avidin-biotin-peroxidase complex x400); b) Details of negative CD56 expression on the histological sample for the same case (avidin-biotin-peroxidase complex x300).

The expression of each immunomarker did not result in any discordant expression between cytological and histological samples per each case analyzed. In detail CD56 expression was completely negative in 48 out of 50 PMs (96%—Fig 3A and 3B) whereas the remaining 2 FVPC showed weak and focal expression (5% of cells were positive).

**Fig 4:**
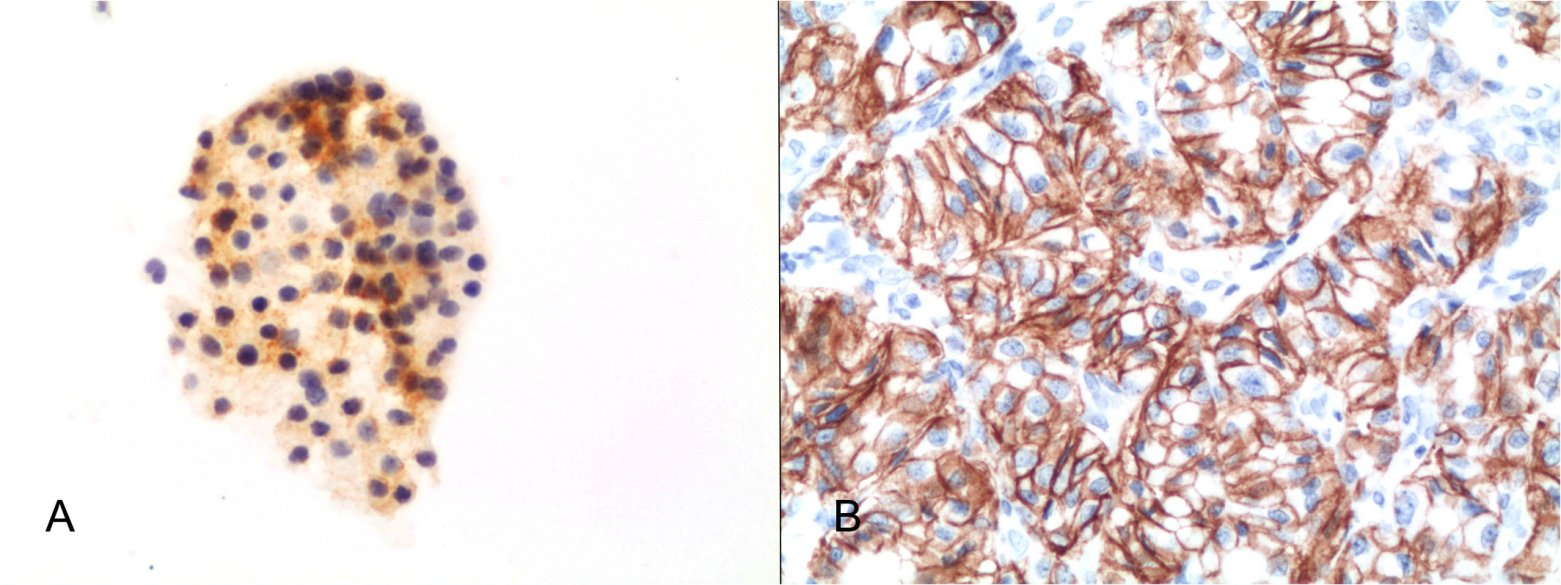
Details of cytoplasm-membranous CD56 positivity in a benign lesion diagnosed as BN (avidin-biotin-peroxidase complex x400); b) Details of diffuse cytoplasm and membrane CD56 expression on the histological sample for the same case (avidin-biotin-peroxidase complex x300).

On the other hand, the cells in BNs showed a moderate-strong membranous and cytoplasm staining for CD56 in 24 out of 35 (68.5%—Fig 4A and 4B) even though CD56 resulted in a statistical significance (p-value <0.00001, O.R. 523.636, 95% CI from 10.7389 to 255.3).

Galectin-3 revealed strong cytoplasm expression in 96% of the PMs whilst 71.5% of the BNs were negative (p<0.0001 O.R. 600, 95% CI from 12.1950 to 295.202). The two PMs with Galectin-3 negative expression had a histological diagnosis of encapsulated FVPCs. Interestingly, these two cases were negative for both HBME-1 and Galectin-3 with CD56 positivity corresponding to an IHC pattern more frequently encountered in a BN diagnosis. The expression of HBME-1 was found in 45 positive PMs out of 50 (90%) with 77.2% of the BNs showing a negative expression (p<0.0001 O.R. 303.750, 95% CI from 9.0128 to 102.3704). Furthermore the different histotypes (PTCs, encapsulated FVPCs, infiltrative FVPCs and TCVs) did not show any statistical significant discrepancy in the IHC expression (data not tabled). Table 3 highlighted the statistical value for each single immunomarker.

**Table 3:** Descriptive statistics for each immunocytochemical marker in the cyto-histological series.

	Sens.	Spec.	Diagn. Acc.	OR (95% CI)	p-value
**CD56**	96%	69%	85%	52.4 (10,7–255,3)	p < 0,0001
**HBME-1**	90%	77%	86%	38.4 (9–102.3)	p < 0,0001
**Galectin-3**	96%	71%	86%	60 (12.2–295.2)	p < 0,0001

Sens: Sensitivity; Spec: Specificity; Diagn. Acc.: Diagnostic accuracy; OR (95% CI): Odds ratio (95% Confidence intervals). A p-value less than 0.05 is considered significant.

In Table 4 we analyzed the use of these immunomarkers as a panel including two or all the three of them. According to the literature reporting that the positive expression of HBME-1 and/or Galectin-3 are found to be highly associated with PTCs and its variants, we correlate the combination of each of the two immunomarkers with CD56.

**Table 4:** Concordant staining score for CD56, HBME-1 and Galectin-3 double and triple ICC combinations with histological follow-up^§^.

	HBME-1 / Galectin-3 (80 cases)		CD56 / HBME-1 (72 cases)		CD 56 / Galectin-3 (70 cases)		CD56 / HBME-1 / Galectin-3 (68 cases)	
	H- / G-	H+ / G+	C+ / H-	C- / H+	C+ / G-	C- / G+	C+ / H- / G-	C- / H+ / G+
**Benign**	23 (29%)	7 (8%)	20 (28%)	3 (4%)	18 (26%)	4 (6%)	18 (27%)	3 (4%)
**Malignant**	3 (4%)	47 (59%)	1 (1%)	48 (67%)	1 (1%)	47 (67%)	1 (1%)	46 (68%)

(C+) CD56 positive; (C-) CD56 negative; (H+) HBME-1 positive; (H-) HBME-1 negative; (G+) Galectin-3 positive; (G-) Galectin-3 negative.

^§^Data for the discordant ICC cases were reported in the supplementary material (S1 Table).

Although we analyzed all the possible ICC combination (data also in S1 Table), we focused the table on the figures including the concordant expression of HBME-1 and Galectin-3, the cases combining CD56 with each of HBME-1 and Galectin-3 and the entire panel with the most frequent malignant correlation. These specific combination were found in the majority of our cases (ranging from 85% to 82% respectively) and they had a significant value (p<0.0001). Additionally the application of the entire panel showing concordant positive HBME-1 and Galectin-3 and negative CD56 was found in 80% of the cases and as stated in Table 5, it represented the best sensitivity (98%), specificity (86%) and diagnostic accuracy (94%).

**Table 5:** Descriptive statistics for immunocytochemical markers used in double and triple combinations in the concordant cyto-histological series^§^.

	Sens.	Spec.	Diagn. Acc.	OR (95% CI)	p-value
**HBME-1 / Gal-3**	96%	77%	89%	51.5 (12.2–217.6)	p < 0,0001
**CD56 / HBME-1**	98%	83%	93%	320 (31.4–3264.5)	p < 0,0001
**CD56 / Gal-3**	98%	82%	93%	211.5 (22.1–2022.1)	p < 0,0001
**CD56 / HBME-1 / Gal-3**	98%	86%	94%	276 (27–2831)	p < 0,0001

Sens: Sensitivity; Spec: Specificity; Diagn. Acc.: Diagnostic accuracy; OR (95% CI): Odds ratio (95% Confidence intervals). A p-value less than 0.05 has been considered significant.

^§^Data for the discordant ICC cases were reported in S1 Table

Moreover, the descriptive statistics for different couple of ICC markers and for the entire panel made up of the three markers displayed that the best combination of two immunomarkers should include CD56 coupled with Galectin-3 or HBME-1 (Table 3).

## Discussion

In the present pilot study we seek to ascertain the efficacy of CD56 in discriminating between benign and malignant thyroid FNAC specimens and its role as an additional marker in the panel made up of HBME-1 and Galectin-3 as previously analyzed in some papers published by our group [12, 15, 16].

The diagnostic accuracy of FNAC in achieving a correct diagnosis of PTC has been definitely stated in literature with minimal inter-observer variability [1, 6]. However, the lack of papillary structures, nuclear pseudo-inclusions or focal nuclear pleomorphisms/atypia may lead to confusion and diagnostic dilemma. On the other hands some morphological similarities between benign hyperplastic papillary lesions and PTCs can also be observed and caused diagnostic errors on both FNACs and histological specimens [7, 8, 30–31]. Finally, severe Hashimoto’s thyroiditis may induce nuclear severe atypia, chromatin clearing and even nuclear grooves which may fall short of a correct diagnosis [32].

Thus, the high sensitivity of FNAC in the detection of malignancies is counter-parted by the slightly lower specificity which paved the way for the enthusiasm toward the application of ancillary techniques (ICC and molecular markers) in the discrimination between malignant or benign thyroid lesions as highlighted in the meta-analysis authored by Correia-Rodrigues et al [13].

Consistent with these new insights, among the outstanding variety of markers, several papers observed the highest diagnostic accuracy when HBME-1 and Galectin-3 were performed [13–16, 30–31, 33–36]. In fact, as underlined by Nasr et al and our previous publications, HBME-1 has proven to be the most sensitive and specific marker in 96% of the PTC with only 7% of false positive cases [12, 15, 35]. This evidence recommends caution since HBME-1 positivity has been observed also in Hashimoto thyroiditis and hyperplastic nodules.

Not only did some authors assess the useful role of HBME-1 expression in PTCs but also they strongly suggested that the combination of more than one immunomarker (i.e. HBME-1 and Galectin-3) displayed a higher diagnostic accuracy [12–16, 20, 22, 25–26, 33, 36]. This latter choice is driven by the valuable results provided by both our group and several authors concerning the high positive predictive value (ranging from 78%-100%) of Galectin-3 [12, 15, 34, 36]. Besides, Galectin-3 expression in thyroid carcinoma has been linked with aggressive clinical features whilst negative Galectin-3 carcinomas seem to present a slightly more favorable course with fewer lymph node metastases [34]. Nevertheless up till now, none of these two immunomarkers alone has shown to be the “magic” single antibody of malignancy; in fact Bartolazzi et al reasoned that 25% of the cytological follicular neoplasms (FN) with Galectin-3 positivity resulted in histological benign diagnoses [36]. Therefore, these issues gave rise to the search for new single “marker of malignancy” so that its diagnostic accuracy might achieve more pervasive results. Scant histological studies have suggested that, among them, CD56 may be candidate to be a promising novel marker of thyroid epithelial neoplasms especially when carried out in IHC panel [21–26, 37].

CD56 (an isoform of N-CAM), member of the immunoglobulin superfamily showing 5 extracellular immunoglobulin and two fibronectin type III domains, is catalogued as a neural cell adhesion molecule especially in NK cell-target cell interactions [21]. CD56 is well known to be expressed in neural, mesenchymal tissue but also in normal endocrine cells, namely benign follicles, even though its significance is not yet fully understandable in these latter cells [21–26]. Some previous studies argued that differences in the CD56 expression may affect migration of tumor cells; Kim et al reported that Homeobox 9 (a transcription factor) regulates transcription of CD56 in PTCs and Scarpino et al described that CD56 in PTCs may cause down-regulation of vascular endothelial growth factors D and C (VEGF-D and VEGF-C) which stimulate lymphangiogenesis [23, 38].

Few previous studies performed on thyroid histological samples proved that CD56 is a highly promising immunomarker showing positive expression in the majority of normal thyroid tissue and benign lesions including goiter, Grave’s disease, Hashimoto thyroiditis but with a negative pattern in PTCs (and its variants) [21–26, 37].

In fact, in two distinct series Scarpino et al and El Atti et al had already described both the low expression of CD56 in PTCs and its high specificity when used alone or in an immunopanel [22–23]. All the authors pinpointed diffuse and strong CD56 positivity in their benign lesions counter-parted by negative expression in at around 83–100% of the PTCs depending on the different series [22–23].

Supporting this evidence, El Demellawy et al reported a series of 175 thyroid cases (including both non neoplastic and neoplastic entities) in which CD56 was expressed in all their benign lesions whilst it was negative in all PTCs when a 10% positive cut-off was adopted [21]. In our experience, 96% of the malignant cytological cases had negative CD56 expression underlining the minimal number of false positive PMs on cytological samples which traced the figure found by Park et al in their histological PTCs [37]. In agreement with the results discussed by El Atti et al and El Demellawy et al, we did not find any statistical difference between CD56 expression in PTC, TCV and FVPC (data not tabled). This observation encourages its application, as a valid support for suggesting malignancy also in the FN and SM categories, as several of them resulted in a histological FVPC.

In contrast with the data from El-Demellawy et al describing a diffuse CD56 positivity in all their benign categories, we reported that 31.5% of the BNs resulted CD56 positive and, on the other hand, two PMs were CD56 negative. As emerged from literature, this different range of positivity may be related to the quantitative evaluation of membranous and/or cytoplasm CD56 positivity. [19]. Inasmuch as differences in the chosen cut-off may lead to false-negative results, we adopted the intermediate value of moderate and strong expression in 30% of the follicular or malignant cells; an interesting data is that all our PMs with CD56 negative expression did not show any stained cell.

Furthermore, we compared CD56 expression with HBME-1 and Galectin-3 in order to seek a possible additional marker for a valuable ICC panel.

Our previous published data for HBME-1 and Galectin-3 demonstrated an overall diagnostic accuracy spanning from 81 through 92% in cases with concordant positive panel [12]. As suggested by the previous study, the concordant positive panel allowed the recognition of only 77% malignant outcome [12]. Those data may therefore confirm that the additional use of another marker, such as CD56 positive expression in benign lesions, could correctly identify the remaining 23% of the cases [12]. Our yields for each of the three antibodies proved that the application of an immunopanel is the best choice for a correct cytological diagnosis of the majority of lesions. In fact, in the present series the concordant panel made up of HBME-1 and Galectin-3 assessed 89% diagnostic accuracy which was slightly lower when compared with a panel made up of the three immunomarkers (94% diagnostic accuracy). Although the use of all the three immunomarkers may audit excellent result, in term of cost-effectiveness, our analysis noticed that the best solution seems to be the combination of two immunomarkers made up of CD56 with HBME-1 or Galectin-3. On this hand, as recently published by Pusztaszeri et al, CD117 seemed to be another valuable marker with negative expression for PTCs so that it may represent a valid alternative to CD56 or it may be included in the panel [19].

We are conscious that the bias of our study is in the limited and selected categories of samples mainly ascribed to the use of a prospective series. Nonetheless, our aim was pointed to verify the validation of the role of CD56 in the two categories of BNs and PMs with surgical follow-up excluding in this first study the category of indeterminate lesions which may not be significantly useful for a pilot study of ICC validation.

To the best of our knowledge, CD56 has been mostly studied with formalin-fixed paraffin embedded material, conventional smears [21–26, 37]. Our study is the largest series looking for the CD56 validation as a single marker on cases performed only on LBC as long as the series proposed by Pazaitou et al showed a cytological split sample method followed by the application of an ICC panel (including also CD56) only on the residual material [39]. Although some conflicting opinions and controversial data regarding the efficacy of LBC, we endorse several positive aspects in term of cost-effectiveness, time-sparing, and including also a feasible and reliable application of ancillary techniques (including CD56) [15–16,39–41]. Even though the application of ancillary techniques (ICC or molecular testing) may result in additional costs, their aid in achieving conclusive cytological diagnoses might reduce the number of unnecessary thyroidectomy which oblige to a lifelong drug replacement.

In spite of the well known invaluable role of cell-blocks, we decided to carry out ICC on LBC mainly based on two reasons: firstly our long-standing use of ICC on LBC and secondly because in our personal experience with ICC on cell-blocks we reported some cases with contradictory results (false positive or false negative) attributed to fixation which were not encountered on LBC [12, 15, 16, 40].

Eventually our preliminary results with CD56 emphasize that it may be a reliable marker in ruling out PTCs and its variants. Taking together these data, even though the morphological features represent the gold standard for the majority of thyroid FNAC, suggest that the application of the immunopanel made up of HBME-1, Galectin-3 and CD56 might represent a useful aid in identifying all PMs (including the different variants of PTCs) especially in the category of “suspicious for malignancy” including a significant number of PTCs or FVPCs with subtle malignant features and without the pathognomonic PTC nuclei. In conclusion, our findings suggest a possible role of CD56 as a valuable marker of malignancy in combination with HBME-1 and Galectin-3 so that further studies may evaluate and extend its application also in the worrisome category of follicular patterned lesions and suspicious for malignancy in which the use of morphology alone is no longer the unique source for achieving a correct definitive diagnosis.

## Supporting Information

S1 Table Additional ICC data. (XLS)
